# Quality control of protein reagents for the improvement of research data reproducibility

**DOI:** 10.1038/s41467-021-23167-z

**Published:** 2021-05-14

**Authors:** Ario de Marco, Nick Berrow, Mario Lebendiker, Maria Garcia-Alai, Stefan H. Knauer, Blanca Lopez-Mendez, André Matagne, Annabel Parret, Kim Remans, Stephan Uebel, Bertrand Raynal

**Affiliations:** 1 Lab of Environmental and Life Sciences, University of Nova Gorica, Vipava, Vipava, Slovenia; 2 Protein Expression Core Facility, Institute for Research in Biomedicine (IRB Barcelona), The Barcelona Institute of Science and Technology, Barcelona, Spain; 3 Protein Purification Facility, Wolfson Centre for Applied Structural Biology, Edmund J. Safra Campus - The Hebrew University of Jerusalem, Jerusalem, Israel; 4 European Molecular Biology Laboratory (EMBL), Hamburg Outstation, Hamburg, Germany; 5 Biochemistry IV - Biopolymers, University of Bayreuth, Bayreuth, Germany; 6 Protein Production and Characterization Platform, Novo Nordisk Foundation Center for Protein Research, Copenhagen, Denmark; 7 Laboratory of Enzymology and Protein Folding, Centre for Protein Engineering, Department of Life Sciences, University of Liège, Building B6C, Allée du 6 Août, 13, Liège, Belgium; 8 Protein Expression and Purification Core Facility, EMBL Heidelberg, Heidelberg, Germany; 9 Charles River Laboratories, Beerse, Belgium; 10 Institut Pasteur, Plateforme de Biophysique moléculaire, Department of Structural Biology and Chemistry, Paris, France

**Keywords:** Proteins, Analytical biochemistry, Molecular biophysics

## Abstract

Proteins and peptides are amongst the most widely used research reagents but often their quality is inadequate and can result in poor data reproducibility. Here we propose a simple set of guidelines that, when correctly applied to protein reagents should provide more reliable experimental data.

There have been several publications over the last decade highlighting the problems of irreproducibility in preclinical research over a wide range of scientific disciplines (see ref. ^1^ for a discussion of the many facets of this problem and ref. ^2^ for a collection of commentaries and analyses for different research sectors). Other reviews have attempted to quantify the economic cost dimension represented by data irreproducibility^3^, focusing on specific reagents widely used by the scientific research community such as antibodies^4^. These reports make uncomfortable reading for researchers, who by training are indeed aware that reproducibility is a critical issue that needs to be tackled^5^. The problem is openly acknowledged by both funding bodies^6^ and journals^7,8^. Thus far, however, the issue appears to have been addressed on a field-by-field basis rather than through a community-wide effort.

Although purified proteins are used in numerous fields of research, no clear standard for the quality control (QC) of protein reagents currently exist and those that do exist are vastly under-utilized. These controls however should be deemed essential from a scientific point of view, to allow the identification of poor quality or artefactual research as early as possible to limit snowball effects; whereby a published paper can rapidly spawn a huge number of secondary papers and citations even when the original data are not reproducible. Although there have been many reports (see e.g., refs. ^9–12^) describing the effects of poor protein quality on the validity and reproducibility of experimental data, to date there has been little visible response to this specific problem from the research community.

The use of poor quality peptides, proteins and antibodies as experimental reagents impacts both the quality and cost of research carried out using these reagents. One estimate^3^ puts a figure on the level of irreproducible preclinical experiments in the US (using 2012 data) at fifty percent, equating to a staggering economic cost of $28 billion per annum in the US alone, of which thirtysix percent ($10.4 billion worth of research) was directly attributed to poor quality ‘biological reagents and reference materials’. At present we are aware of only very few journals where there is a requirement for authors to include QC data for the proteins used as ‘reagents’ in their studies. This situation appears to be in direct contrast to e.g., the high standards of statistical analyses and declarations of statistical compliance required in articles submitted to high-end journals when presenting genomic, proteomic and structural data^13^. With the aim of addressing this obvious imbalance, and in response to the problem of data reproducibility when protein reagents are involved, a working group comprised of members of both the ARBRE-MOBIEU and the P4EU networks produced a list of recommended tests (QC Guidelines – reported in Supplementary Note 1 and accessible at https://p4eu.org/protein-quality-standard-pqs or https://arbre-mobieu.eu/guidelines-on-protein-quality-control). These guidelines were developed with reference to the available literature^12,14^ and the extensive professional experience of the working group members, to aid in the validation of protein samples used in biological research. They have been embraced by a wide community of specialists (a full list of these researchers can be found on ARBRE-MOBIEU and P4EU website) and comprise three parts: (1) minimal information, (2) minimal QC tests, and (3) extended QC tests. We propose a list of minimal QC tests that are based on simple experimental methods that are widely available (Supplementary Table 1 and Supplementary Note 1, Supplementary Figs. 1–7). Together with this minimal information, we feel that these or similar disclosures should become compulsory documents in any submission to scientific journals when using protein/peptide reagents. While generally considered complementary, extended QC tests may be considered essential when using the proteins in specific experimental downstream applications. Our protein QC guidelines are summarized described below and schematically illustrated (Fig. 1).

**Fig. 1:**
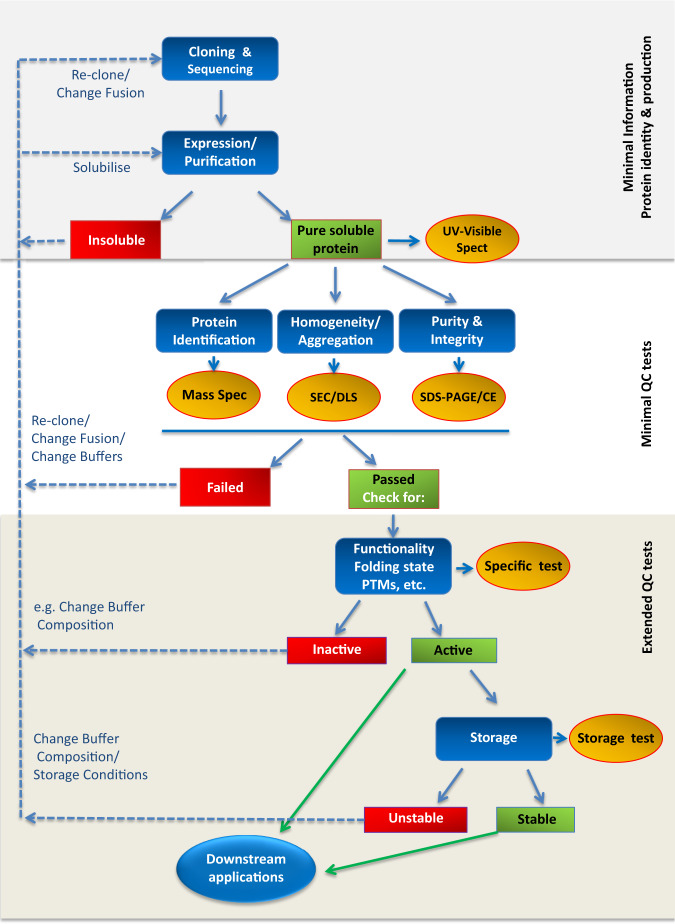
Protein reagents: evaluation of Protein Identity, Preparation and Quality Control. Blue icons indicate process steps, whereas yellow icons display quality control requested experiments. The actual DNA sequence of the clone must be verified for its identity/correctness (correspondence to original clone, no mutations) before starting its expression. Following purification, the identity of the protein must be confirmed (by Mass Spectrometry), its purity and integrity evaluated (SDS-PAGE/CE), and its homogeneity (i.e., size distribution/aggregation state) checked to assess size distribution (i.e., monodispersity/polydispersity). The most accessible tests are reported (SEC, DLS), alternatives can be found in the guidelines. If all minimal QC tests are passed, proteins should be tested for further properties, e.g. their functionality or their folding state before being used as reagents. Further analyses are necessary for specific protein applications, as it can be the case of DNA contaminations (extended tests described in the on-line guidelines/SN1), and to evaluate the possibility to store the protein. If proteins do not pass any of the check steps, their production/storage process should be optimized. Summarizing, the minimum QC relies on three parameters (i.e., identity, purity, integrity and homogeneity) requiring three (first-line) analytical methods only. As indicated, it is possible to choose between alternatives: SDS-PAGE or CE, analytical SEC or DLS. The requirement in terms of protein is roughly 100 μg [SDS-PAGE, 10 μg (Coomassie blue staining); Mass Spectrometry, 60 μg; Analytical SEC, 30 μg (for Dynamic Light Scattering, 20 μg, the sample can be recovered)]. UV-Visible spectrophotometry is advised since the protein is recycled and several pieces of information can be rapidly collected (Supplementary Note 1).

## Minimal information

1. For recombinant proteins, the complete sequence of the construct used in the reported experiments should be made available and we highly recommend confirming the sequence after cloning by sequencing to avoid wasteful production trials.
2. Expression, purification and storage conditions should be *fully* described such that they may be accurately reproduced in any laboratory.
3. The method used for measuring the protein concentration should be given

## Minimal QC tests

1. Protein purity should be assessed by any of common techniques such as SDS-PAGE, Capillary Electrophoresis (CE), Reversed Phase Liquid Chromatography (RPLC). Mass Spectrometry (MS) and RPLC help to detect the presence of contaminating proteins, sample proteolysis and minor truncations.
2. Homogeneity/dispersity refers here to the size distribution of the protein sample, which can generally be correlated with oligomeric state (monomer, dimer etc.) or the presence of aggregates. Whereas poly-dispersity is not per se an indication of instability, preparations showing the presence of ‘incorrect’ oligomeric states or higher order ‘aggregates’ suggest that the protein may not be in an optimal/functional state. This can have a dramatic effect on the results of experiments to determine e.g. enzyme kinetics and protein-ligand interactions, essentially as a result of an overestimation of the concentration of active protein. Protein homogeneity/dispersity may be assessed by Dynamic Light Scattering (DLS), size exclusion chromatography (SEC) or, preferably, by SEC coupled to multi-angle light scattering.
3. The identity of a sample can be confirmed using either ‘bottom-up’ MS (mass fingerprinting or tryptic digests) or ‘top-down’ MS (by measuring intact protein mass). The former will confirm that the correct protein is being used and not e.g. a host protein of similar mass that has been purified in error. The latter will confirm the identity of the protein and will also indicate whether it has suffered any proteolysis during purification (intactness/micro-heterogeneity).

## Extended QC tests

In addition to this short list of minimal/essential controls, other techniques are recommended to further characterize protein samples and their suitability as experimental reagents, for instance the folding state of proteins and the specific activity of enzymes. Proteins produced in *Escherichia coli* that are destined for use in experiments with cultured cells should be tested for the presence of lipopolysaccharides/endotoxins and UV spectrophotometry is mandatory for DNA/RNA binding proteins.

Examples in which protein quality assessment resulted in improvements of sample quality with critical impact on downstream experimental results are presented in supplementary information (Supplementary Note 2, Supplementary Figs. 8–12). The results of a large scale survey among users who volunteered applying the guidelines in their routine experiments has also been carried out^15^.

## Conclusions

In our experience, the application of the limited number of simple QC tests suggested above provides reliable indicators of the quality of the protein employed as experimental reagents, and yields more reproducible results in downstream applications. We believe that their implementation and the public availability of such QC data could therefore significantly increase the level of confidence in the published data resulting from the use of protein reagents, as well as the ability to reliably reproduce the experimental data.

This condition, which should ideally be the norm, is in reality challenged by several factors as reported in a recent survey^5^. Selective reporting, insufficient availability of raw data and the paucity of information in many ‘Materials and Methods’ sections are all factors which contribute to create opacity. The decline of the essential materials and methods sections of published papers dates back, understandably, to the times when many journals were available only in print and the pressures to minimize the sizes of submitted papers. With the advent of on-line publishing it is time to advocate the (re-) integration of these essential sections to their former status to allow other researchers to reproduce the data therein without resorting to making contact with the authors. Although this effect has been partly mitigated by the current availability of Supplementary Data sections in many on-line journals, the presented data often falls short of a full description of the experimental conditions used and often lacks any form of QC data relating to protein quality. The present interest of Editors for the systematic storage of (raw) data [https://www.springernature.com/gp/open-research/open-data/practical-challenges-white-paper] should consider also the inclusion of this methodological data.

We suggest that implementation of guidelines for protein quality evaluation should be considered an entry point towards the development of improved and ideally compulsory reporting practices of data obtained with protein reagents. It is our contention that ‘Supplementary Data’ sections should also contain details of the QC tests performed on any protein/peptide reagents used in a study, independent of the source of the protein reagent (commercial vendors or purified in an academic lab), in order to give referees and readers an indication of the quality of the materials being used to derive any given data set. To this effect, we suggest the development—in co-operation with journal editors—of a standardized form for QC reporting and annotation for authors to complete during the submission process. A model of such a checklist is illustrated in Supplementary Table 1 and could be made available to referees and editors but also published in the supplementary material to allow reader scrutiny. Finally, all the stakeholders—scientists, editors and funding agencies—will profit from improving data reliability and reproduction by means of systematic and accurate reagent QC. Such practices should minimize the wasteing of time and resources and, in addition, favor future metadata analysis.

## Supplementary information

Supplementary Information
