# Supplementary material for: Quality control of protein reagents for the improvement of research data reproducibility
Source: Nat Commun. 2021 May 14;12:2795. doi: 10.1038/s41467-021-23167-z (PMC8121922; doi:10.1038/s41467-021-23167-z)
Supplement: Supplementary file 1 — Supplementary Information [file 41467_2021_23167_MOESM1_ESM.pdf]

Supplementary Information

Supplementary Table 1. Check-list for protein quality control

The minimal requirements necessary for evaluating the protein quality can be obtained by a combination of alternative methods. Here the thresholds for the experiments indicated in the attached flowchart (Fig. 1) are reported. Their complete list, with practical tips for particular methods, is available in the on-line guidelines, reported here as Supplementary Material 1, in which is also explained how to assess the experimental results.

We highly recommend to record a full UV absorbance spectrum from 200-340 nm directly after finishing purification, i.e. before carrying out any QC test, as this spectrum will give information about the concentration of the sample, the presence of nucleic acids, and aggregation (for details see Supplementary Material 1). Other methods, such as chromatographic analytical methods to confirm sample heterogeneity, can represent more accessible alternatives to golden standards.
Depending on the end-use of the protein, attention should also be paid to the sample storage conditions. Thus, it is highly advisable that stored samples are re-tested for integrity and dispersity before subsequent use.

	Scope	Information provided or test applied	Expected Thresholds values Requirements	Results			
				Passed	Failed	Not performed Not applicable	Data provided (Y/N)
Minimal information	Provide general information about the protein	Protein name	Corresponding GenBank/UniProt reference				
		Full primary structure	Fully translated sequence given or derivable from given data				
			Gene sequence confirmed by sequencing				
		Expression vector & host strain / source	Details given are sufficient to accurately reproduce data				
		Expression & purification protocol	Details given are sufficient to accurately reproduce data				
		Protein concentration	Determination method must be described				
		Storage conditions	Details given are sufficient to accurately reproduce data				
Minimal QC tests	Check purity	SDS-PAGE (R-250 coomassie -silver staining)	correct molecular mass ± 5% with respect to the theoretically calculated molecular mass				
			absence of contaminants and / or degradation if no more than 2 % total contaminants				
	Check identity	intact MS (top-down)	correct molecular mass ± 1 Da with respect to the theoretically calculated molecular mass.				
		bottom-up MS	100% Sequence agreement with the reference sequence (if not, give sequence coverage)				
	check homogeneity / aggregation state	Analytical SEC or SEC -MALS	no aggregation if (i) no peak in the void volume, (ii) a single, well-defined, symmetric peak, representing a homogeneous species, (iii) no further peaks				
			other contaminants/ degradation products observed?				
			if coupled to a MALS system: the determined molecular masses match the theoretical values				
		DLS	homogeneous solution if detection of only one peak with polydispersity less than 20% of the peak				
		mass photometry	single, well-defined peak matching the expected molecular mass of the protein/protein complex				

**Extended QC tests**	**general QC test by UV spectroscopy**	UV spectrum from 200-340 nm	absence of nucleic acid contaminants indicated by $A_{260}/A_{280} < 0.6 \pm 0.1$				
			no aggregation, indicated by $A_{340}/(A_{280}\text{-}A_{340})*100 <2$				
	**check homogeneity, e.g. in terms of charge variants, different oligomeric states, post-translational modifications, shape variants, …**	IEX (-MALS)	homogeneous soluton (in terms of one defined charge species and /or homogeneous PTMs) indicated by a major, well-defined, symmetric peak; minor peaks possible; if coupled to MALS: correct molecular mass of each peak				
		HIC (-MALS)	homogeneous solution (one defined shape variant) indicated by a major, well-defined, symmetric peak; minor peaks possible; if coupled to MALS: correct molecular mass of each peak				
		IEF	homogeneous solution (in terms of one defined charge species) indicated by a major band at expected pH, and possible minor bands close to the major one for recombinant proteins from eukaryotic systems				
		other:					
	**conformational stability / folding state**	CD	spectrum is consistent with the expected secondary structure elements				
			protein is folded				
		DSC	T_m matches reorted values				
		NMR	protein is folded (indicated by a 1D ^{1}H spectrum)				
		FTIR	position of amide I band consistent with presence of expected secondary structure elements				
		other:					

Extended QC tests	check protein competent fraction	determine specific activity	matches reported values				
		check interaction proterties	matches reported values				
		other:					
	endotoxin content optimization of storage conditions	LAL	less than 0.5 endotoxin units per milliliter (EU/ml), corresponding to 0.05 ng endotoxin/mL of solution				
		other:					
		DSC	best buffer results in highest T_m				
		DSF	best buffer results in highest T_m				
		DLS	good buffers result in homogeneous samples showing no aggregation				
		other:					
	Batch-to-batch consistency	CD	spectrum identical to reference spectrum (recorded with batch "0")				
		NMR	spectrum identical to reference spectrum (recorded with batch "0")				
		other:					

Supplementary Note 1. Quality control of purified proteins – Complete Guidelines

Please note that these will be frequently updated online at: https://p4eu.org/protein-quality-standard-pqs or https://arbre-mobieu.eu/guidelines-on-protein-quality-control

Best practice recommendations

Two pan-European networks, the *Association of Resources for Biophysical Research in Europe* (ARBRE-MOBIEU) and the *Production and Purification Partnership in Europe* (P4EU) have produced a joint initiative on recombinant protein quality. We aim to develop a minimal reporting standard/best practice for the quality control of recombinant proteins to ensure that the input material used in biological, biophysical and biochemical research is of high quality. This condition, in turn, will result in more reliable and reproducible final data. The prescribed tests must be simple to perform using standard laboratory equipment while still producing data acceptable as admission criteria for biophysical or structural biology labs.

Below you find some recommendations for what we propose as (1) minimal information, (2) minimal quality control parameters and (3) extended quality control parameters/standards. Example of results with the most used methods are presented.

1) Minimal information to provide in publications[1(see appendix)]

The aim is to provide sufficient information on the **protein identity, expression and purification parameters** such that the experiment can be replicated reliably in any laboratory.

- **Protein name and full primary structure**, by providing a NCBI (or UniProt) accession number, cloning strategies, and the source of the DNA (species).
- **Expression vector and host strain**, including the tags and cleavage sites used, accompanied by the full amino acid sequence of the final protein, or sufficient details to derive the full amino acid sequence of the final protein.
- **Expression and Purification protocol**, namely the detailed description of all the protein production steps.
- **Protein concentration** (specifying the method used for quantification and the molar extinction coefficient at 280 nm, if applicable. Extinction coefficient can be obtained for any sequence by using open access resources -https://web.expasy.org/protparam/).
- **Storage conditions**, i.e. final buffer composition (pH, buffers, salts and additives), storage temperature and, where applicable, freezing or lyophilization conditions.

2) Minimal quality control tests that should be performed on protein samples

- **Purity** [2]**:** checked by SDS-PAGE, Capillary Electrophoresis (CE) or Reverse-Phase-HPLC (RP-HPLC). The objective here is to assess the presence (and level) of contaminants using techniques that are available to most laboratories.

- **Homogeneity (Size distribution/aggregation state)**: checked preferably by Size Exclusion Chromatography (SEC) [3] and/or Dynamic Light Scattering (DLS) [4], or by Size Exclusion Chromatography in combination with Multi Angle Light Scattering (SEC-MALS), Field Flow Fractionation (FFF) or Field Flow Fractionation in combination with Multi Angle Light Scattering (FFF-MALS) or Analytical Ultracentrifugation (AUC). The objective is to assess if the sample has a tendency to form aggregates in the condition used to purify it, and to assess the potential for oligomerization of the protein sample.

- **Identity and integrity:** checked preferably by intact protein mass [5], peptide mass fingerprinting [6] or Edman sequencing.

3) Extended quality control tests

Depending on the intended use and in addition to the methods listed above, the following methods may also be applicable and are highly recommended, although they are not considered to be as essential as those in section (2)

The first of these tests can be performed simultaneously when determining protein concentration spectrophotometrically (using A_{280nm}) as most instruments will also allow you to collect data over a wide spectrum of wavelengths. This additional data can be very informative.

- **General quality test by UV spectroscopy** [7] between 200 nm and 340 nm to check nucleic acid content and general protein fitness/quality: **Mandatory if protein binds nucleic acids.**

- **Homogeneity** [8]**:** The following techniques are complementary to the ones previously described and can be used to assess other aspects of sample homogeneity (charge variants, conformation variants, post-transcriptional modifications): Isoelectric Focusing (IEF) [9], analytical Ion Exchange Chromatography (IEX) [10] or analytical Hydrophobic Interaction Chromatography (HIC) [11]. Both IEX and HIC can be coupled to MALS analysis.

- **Conformational stability/folding state:** Circular Dichroism (CD) spectroscopy [12], Differential Scanning Fluorimetry (DSF), Differential Scanning Calorimetry (DSC), nuclear

magnetic resonance (NMR) [13], Fourier Transform Infra Red (FTIR). The objective of the measurement is to verify the folding signature.

- **Protein competent fraction** [14] i.e. the relative amount of functionally active protein, measured as specific activity, by active site titration or other suitable methods.

- **Endotoxin content** [15], this analysis is mandatory for protein samples used in combination with cell cultures.

- **Optimization of storage conditions**: Find the best buffer conditions, minimize the formation of protein aggregates, improve solubility and stability. This needs adjustment of several parameters of the sample buffer composition, like: pH, conductivity, presence of detergents, cryo-protectants, chaotropic or kosmotropic additives, co-factors, ligands, others[16]. These conditions can be assessed using automated techniques such as DLS [17] (described above) or thermal shift assay and differential scanning fluorimetry (DSF) assay [18].

- **Batch-to-batch consistency** [19]**:** Mandatory if more than one batch is used. Many factors can affect the quality of your protein and you should never assume that all preps are of equal quality.

APPENDIX

[1]Example of Minimal information to provide in publications

eSpCas9 (including both N and C-terminal NLS sequences) was amplified from plasmid eSpCas9(1.1) (Addgene plasmid 71814) using the high fidelity KOD polymerase (Merck) and the following primers:

fwd: 5’AGGAGATATACCATGGCCCCAAAGAAGAAGCGGAAGG3’
rev: 5’CCACGAAAAAGGCCGGCCAGGCAAAAAAGAAAAAGAAACATCACCATCAC3’

The resulting 4227 b.p. PCR product was DpnI treated to remove the template and purified using Ampure (Beckman Coulter) before In-Fusion (Clontech) into *Nco*I-*Pme*I cut pOPINE (Addgene plasmid 26043). The resulting plasmid will express *eSpCas9* with an N-terminal NLS and a C-terminal NLS-His-tag. The plasmid was fully sequenced before *E. coli* Rosetta(DE3) pLysS cells were transformed with it, and transformants were selected on LB agar plates supplemented with 1% glucose, Carbenicillin (50µg/ml) and Chloramphenicol (35 µg/ml). A starter culture was grown overnight at 37°C with shaking at 250 rpm in LB supplemented with 1% (w/v) glucose, Carbenicillin (50 µg/ml) and Chloramphenicol (35 µg/ml). The culture was then diluted 1:50 in auto-induction TB Overnight Express media (Merck) supplemented with Carbenicillin (50 µg/ml) and Chloramphenicol (35 µg/ml), grown for a further 3 h at 37°C until the OD_{600} reached the value of 0.5, then the temperature reduced to 25°C and the bacteria were grown for a further 24 h. Cells were collected by centrifugation at 5000x*g* for 10 min and the pellets (15 g/L) stored at -80°C before processing. Pellets were lysed in 5 volumes of **Lysis/Loading Buffer (**20 mM TrisHCl, pH 8, 500 mM NaCl, 30 mM Imidazole supplemented with 1 complete EDTA free tablet [Roche] and 1000 Kunits of DNAse [Merck]/10 g of pellet) at

20 Kpsi using a cell disruptor (Constant Systems Ltd. UK). The lysate was cleared by centrifugation for 20 minutes at 30,000x*g* at 4°C and filtration (cut-off 0.4 µm) before loading onto a 5 ml HisTrap HP column (GE Healthcare). After sample loading the column was washed with 10CV of loading buffer before the eSpCas9 was eluted using **Elution Buffer (**20 mM Tris pH8, 500 mM NaCl, 500 mM Imidazole, 2 mM DTT, 10% glycerol). Peak fractions were analyzed by SDS-PAGE before pooling and buffer exchange into **DESALT/Storage Buffer (**20 mM Tris pH8, 200 mM KCl, 10 mM $MgCl_2$) using a HiPrep Desalting column (GE Healthcare). eSpCas9 was then concentrated to a maximum of 3 mg/ml using a ultrafiltration device with a 30 kDa MW cut-off, concentration was assayed using a Nanodrop instrument (Thermo Scientific) and a calculated A_{280} of 0.678 A_{280nm}/mg/ml (or 110230 M^{-1} cm^{-1} calculated using Protein calculator from the Scripps Research Institute-http://protcalc.sourceforge.net/). eSpCas9 fraction was then separated into single-use aliquots that were flash-frozen in liquid nitrogen and stored at -80°C until further use.

[2] **Example: SDS-PAGE**

If SDS-PAGE is the sole method of assessment of sample purity then ideally no other band other than the expected one for your protein of interest should be detected, as illustrated in the Supplementary figure 1. The staining should be chosen according to the amount loaded on the gel in order to be able to detect contaminants of 1% or less of the total protein load. N.B.: the detection limit of Coomassie blue staining is approximately 100ng per band, for reverse zinc staining approximately 10 ng per band, while fluorescent or silver stains have a detection limit of approximately 1 ng of protein per band. In other words, if you load 10 µL of a solution at 1 mg/ml, you will load in total 10 µg of protein. With this quantity of protein loaded you will need sensitivity lower than 100 ng per band in order to detect contaminants and you should therefore use reverse zinc, fluorescent or silver stain to be able to assess contamination. It is also possible to perform TCA or DOC/TCA or acetone precipitation to concentrate the sample ten or more times.

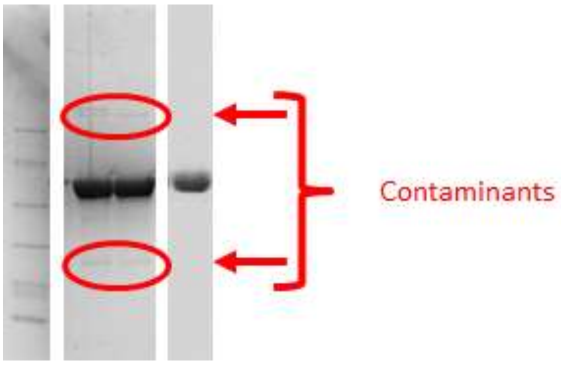

Supplementary Figure 1: SDS-PAGE

Common staining protocols can be found in:

- Quality assessment and optimization of purified protein samples: why and how? Raynal B, Lenormand P, Baron B, Hoos S, England P. *Microb Cell Fact* ***2014; 13:180***.

More details about other techniques can be found in:

- Applications of capillary electrophoresis in characterizing recombinant protein therapeutics. Zhao SS, Chen DDY. *Electrophoresis* ***2014; 35:96–108.***
- Hydrophobic interaction chromatography for the characterization of monoclonal antibodies and related products. Fekete S, Veuthey JL, Beck A, Guillarme D. *J Pharm Biomed Anal* ***2016; 130:3-18.***

[3]**Example of Homogeneity by SEC** If you are using analytical size exclusion chromatography to assess homogeneity, only regular peaks corresponding to the prevalent monomeric or oligomeric species specific for that protein should be detected, and no aggregates should be detected in the void volume of the column situated in a position corresponding to 1/3 of the total volume of the size exclusion column used (e.g. for column with a total volume of 21 ml the void volume is situated at an elution volume of 7 ml). The supplementary figure 2 below shows a good sample (filled dots) with a single, well-defined, symmetric peak, representing a homogeneous species, and a heterogeneous sample containing aggregates (open dots), note the 'aggregate' peak at the void volume of the column (circled) and the asymmetric main peak. SEC can, of course, also be used preparatively at larger scales to select specific oligomeric states e.g. if a dimer is functional in an experiment or assay but a monomer is not the peak corresponding to the size of the dimer may be selected. Please note that after pooling a specifically 'sized' peak any further processes e.g. concentration or buffer exchange may alter the oligomeric state. Pooled fractions from a single peak should preferably be checked again by a further SEC round.

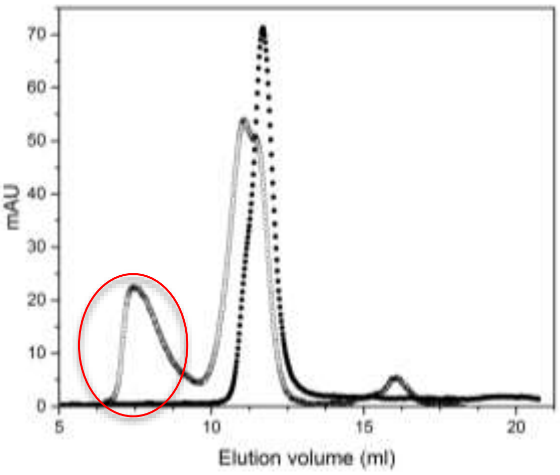

Supplementary Figure 2: Size exclusion chromatography

More detail about Size exclusion Chromatography can be found in:

- Theory and practice of size exclusion chromatography for the analysis of protein aggregates. Fekete S, Beck A, Veuthey J-L, Guillarme D: *J Pharm Biomed Anal* ***2014; 101:161–173.***
- Useful practical information can be accessed in: https://www.gelifesciences.com/en/de/solutions/protein-research/knowledge-center/protein-handbooks and http://wolfson.huji.ac.il/purification/

Analytical SEC-MALS

Same threshold as for analytical SEC, plus the additional value of MALS and RI information. The method is highly sensitive to detect aggregates and it measures the absolute molar mass. Thus, you obtain information about the size and shape of macromolecules in solution (oligomeric conformation of each peak), the total mass of the molecule and the contribution of the mass of both protein and conjugate (carbohydrates, detergents for membrane proteins, others), and non-protein complexes like big polysaccharides, micelles, etc can be detected.

- Determination of molecular masses of proteins in solution: Implementation of an HPLC size exclusion chromatography and laser light scattering service in a core laboratory. Folta-Stogniew E, Williams KR: *J Biomol Technol* ***1999; 10:51-63.***

- Online size-exclusion high-performance liquid chromatography light scattering and differential refractometry methods to determine degree of polymer conjugation to proteins and protein-protein or protein-ligand association states. Kendrick BS et al.: *Anal Biochem* ***2001; 299:136-146***.

- Characterization of proteins by size-exclusion chromatography coupled to multi-angle light scattering (SEC-MALS). Some D et al.: *J Vis Exp* ***2019; e59615***.

[4] **Example: DLS**

If DLS is used to assess the homogeneity of your sample ideally only one species with a low polydispersity (with less than 20% dispersity of the peak) should be detected, and no aggregates should be detectable (as greater than 1-2 percent of your sample). The figure below shows a good sample (Left Supplementary Figure 3) with a single peak and a heterogeneous sample containing aggregates (Right Figure 3).

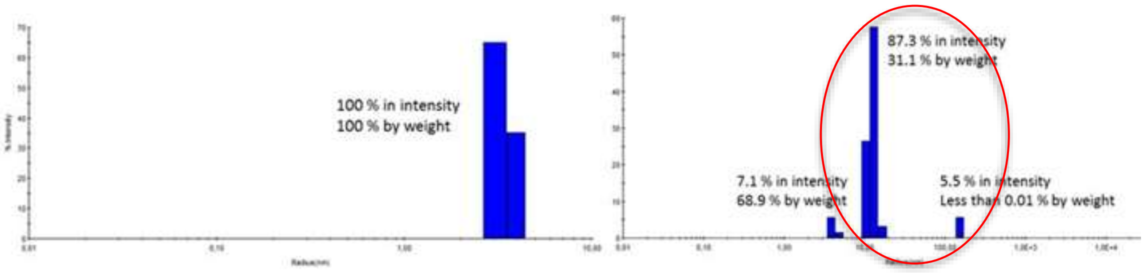

Supplementary Figure 3: Dynamic light scattering analysis

One should remember that the signal is dependent on the size of the detected particle. For example in the right figure the aggregate of 150 nm (right circle) represents more than 5% of signal, however less than 0.01% in weight, it is therefore negligible. On the contrary, the aggregates with an approximate size of 15 nm (central peak) represent nearly 90% of the intensity signal but only 31% of the sample weight. This sample can clearly be defined as non-homogeneous.

More detail about DLS can be found in:

- Dynamic light scattering: a practical guide and applications in biomedical sciences. Stetefeld J, McKenna SA, Patel TR. *Biophys Rev* ***2016; 8:409-427.***

- Dynamic light scattering as a relative tool for assessing the molecular integrity and stability of monoclonal antibodies. Nobbmann U, Connah M, Fish B, Varley P, Gee C, Mulot S, Chen J, Zhou L, Lu Y, Shen F, Yi J, Harding SE. *Biotechnol Genet Eng Rev* ***2007; 24:117–128.***

- Is any measurement method optimal for all aggregate sizes and types? Philo JS. *AAPS J* ***2006; 8:E564–571.***

[5] **Example: Intact mass by MALDI-TOF**

For confirmation of sample identity mass spectrometry is a technique that is now widely available, both in academic institutions and commercially. Mass spectrometry will identify species according to their mass over charge ratio. In the intact mass (also known as 'top-down' MS) example presented here (see supplementary figure 4), the purified protein has an expected mass of 15154.8 Da. Two peaks can be detected: one with a double charge and a ratio of 7578.34 m/z and one with a single charge and a ratio of 15155.56 m/z. Since these two peaks are in good agreement with the expected molecular mass, the produced protein is the expected one and no trace of degradation or contamination can be detected.

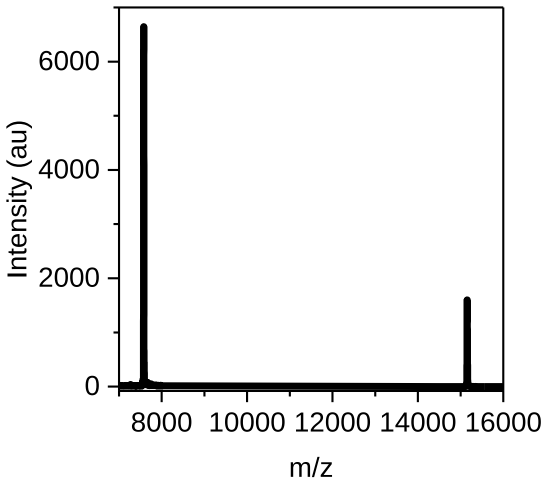

Supplementary Figure 4: Intact Mass spectrum

[6] **Example: tryptic digest/MS**

You may also use 'bottom-up' mass spectrometry (e.g. tryptic digest/MS) to confirm the identity of our protein. This method will also detect (and identify) any contaminating proteins but will not provide information on the integrity of your protein. It can be conveniently performed on samples excised from SDS-PAGE gels.

More detail about mass spectrometry can be found in:

- Analysis of intact protein isoforms by mass spectrometry. Tipton JD, Tran JC, Catherman AD, Ahlf DR, Durbin KR, Kelleher NL. *J Biol Chem* ***2011; 286:25451–25458.***
- Overview of peptide and protein analysis by mass spectrometry. Zhang G, Annan RS, Carr S, Neubert T. *Curr Protoc Protein Sci* ***2010; 62:16.1.1–16.1.30***

[7] **UV absorbance between 240 nm and 340 nm**

As general quality control test, the simplest qualitative test is the measurement of UV absorbance between 240 nm and 340 nm. One of the main advantages is the availability of such equipment in all the biological laboratories. Apart from measuring the concentration by means of the protein extinction coefficient, a full spectrum will give valuable information on the general quality of the preparation as well. It should be stressed that the exact protein buffer needs to be used as a blank in order to avoid errors in the interpretation. A strong signal at 260 nm is usually a sign of nucleic acid contamination. As a general rule the ratio between the absorbance at 260 nm and 280 nm (A_{260}/A_{280}) should give a value close to 0.6 for a good quality protein preparation.

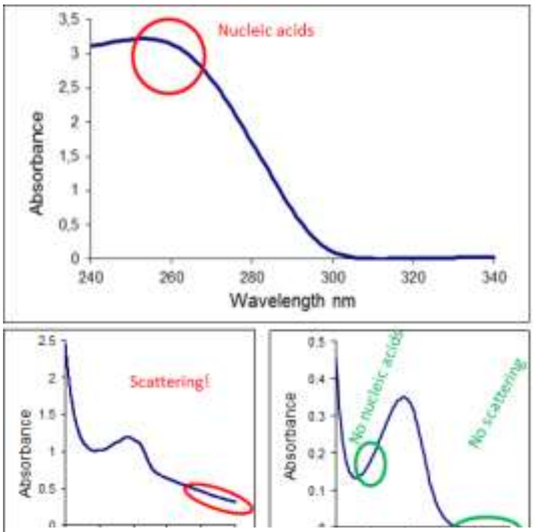

Supplementary Figure 5: Example UV spectrum

Furthermore, a sample that produces significant absorbance that extends past 300nm (usually seen as a slowly decreasing absorbance between 300 nm and 340 nm-see figure) is usually a sign of scattering due to aggregation. (see Supplementary Figure 5). One simple way to confirm scattering is to determine an aggregation index by calculating 100 x $A_{340}/[A_{280} - A_{340}]$. As a rule of thumb, an index lower than 2 would be acceptable for a non-aggregated protein.

More detail about UV spectrometry can be found in:

- Effect of Light Scattering on Ultraviolet Difference Spectra. Leach SJ, Scheraga HA. *J Am Chem Soc* ***1960; 82:4790–4792.***
- Validity of nucleic acid purities monitored by 260nm/280nm absorbance ratios. Glasel JA. *Biotechniques* ***1995; 18:62-3.***
- Dual effects of Tween 80 on protein stability. Wang W1, Wang YJ, Wang DQ. *Int J Pharmaceutics* ***2008; 347:31–38***
- Monodispersity of recombinant Cre recombinase correlates with its effectiveness in vivo. Capasso P, Aliprandi M, Ossolengo G, Edenhofer F, de Marco A. *BMC Biotechnol* ***2009; 9:80***
- [8] Separation techniques: Chromatography. Coskun O. *North Clin Istanb.* ***2016; 3:156–160.***

[9] **IEF** (Isoelectric Focusing): Protein heterogeneity, charge variants. Expected values: a major band at expected pH, and possible minor bands close to the major one for recombinant proteins from eukaryotic systems

- Separations of peptides and proteins. Pergande MR, Cologna SM: *Proteomes* ***2017; 5:E4***

[10]**Analytical IEX** (ion exchange). Scope: Protein heterogeneity, charge variants, impurity. Extremely important for proteins produced in eukaryotic systems). Threshold Value: charge homogeneity. Separation of highly oligomeric conformations. Highly recommended for proteins used in crystallization

Analytical IEX-MALS: Same as analytical IEX, plus the additional value of MALS and RI information. The method is highly sensitive to detect aggregates and it measures the absolute molar mass. Thus, you obtain information about the size and shape of macromolecules in solution (oligomeric conformation of each peak), the total mass of the molecule and the contribution of the mass of both protein and conjugate (carbohydrates, detergents for membrane proteins, others), and non-protein complexes like big polysaccharides, micelles, etc can be detected.

- Coupling Multi Angle Light Scattering to Ion Exchange chromatography (IEX-MALS) for protein characterization. Amartely H et al.: *Sci Rep* ***2018; 8:6907.***
- Ion Exchange chromatography (IEX) coupled to Multi Angle Light Scattering (MALS) – for protein separation and characterization. Amartely H et al.: *J Vis Exp* ***2019; e59408.***

[11] **Analytical HIC** (hydrophobic interaction). Scope: Protein heterogeneity, shape variants, impurity. Threshold Value: shape homogeneity. Separation of highly oligomeric conformations

[12] **Example: Folding state by circular dichroism**

Circular dichroism (CD) looks at the difference in absorption of left- and right-handed light. In the supplementary figure 6, typical curves of both an unfolded protein (plain line) and of a folded protein (dotted line) are presented.

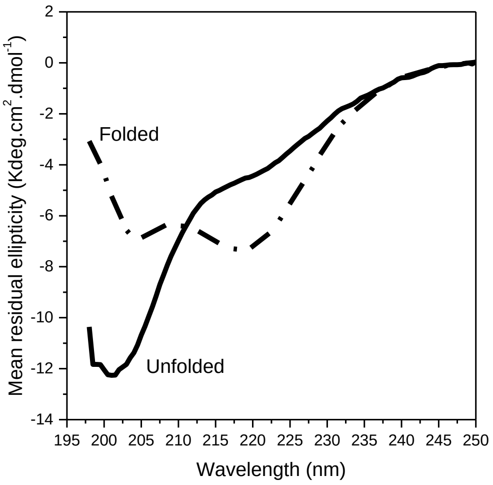

Supplementary Figure 6: Circular dichroism spectrum

More detail about CD can be found in:

- How to study proteins by circular dichroism. Kelly SM, Jess TJ, Price NC. *Biochim Biophys Acta.* ***2005; 1751:119-39.***
- Circular Dichroism: Interpretation and Applications. Woody WR. *Meth Enzymol* ***1995; 246****:34-71*
- http://www.proteinchemist.com/cd/cdspec.html
- Circular Dichroism and the conformational analysis of biomolecules. Fasman GD. *Plenum Press,* ***1997*** *New York and London*
- Circular dichroism and its application to the study of biomolecules. Martin SR, Schilstra MJ. *Meth Cell Biol* ***2008; 84:263-93.***

[13] **Example: Folding state by NMR spectroscopy**

Nuclear magnetic resonance (NMR) spectroscopy is a spectroscopic method to study magnetically active nuclei in an external magnetic field. Measuring a one-dimensional (1D) ^{1}H NMR spectrum of a protein requires little material, is fast and the spectrum clearly indicates if a protein is folded or not. In the 1D ^{1}H spectrum of a well-folded protein (top spectrum) the peaks are narrow and sharp and distributed over a large range of chemical shifts (good signal dispersion); signals can be found especially at ppm values < 0.5 (corresponding to high field-shifted methyl group protons) or > 8.5 (corresponding to down field-shifted amide protons). In contrast, the peaks are broader and not as widely dispersed in the spectrum of an unfolded or partially folded protein (bottom spectrum).

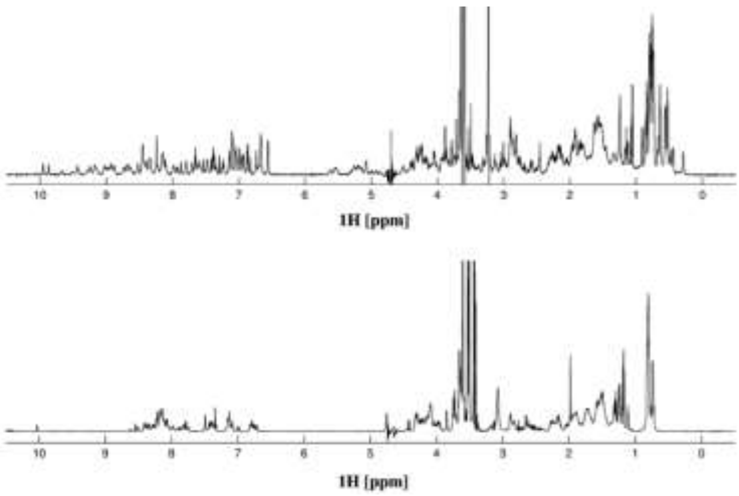

Supplementary Figure 7: NMR spectrum

More details about NMR spectroscopy can be found in:

- Applied NMR Spectroscopy for Chemists and Life Scientists. Zerbe O, Jurt S. *Wiley-VCH Verlag GmbH & Co. KGaA,* ***2014*** *Weinheim, Germany*

[14] **Protein competent fraction**

The objective of this test is to be able to determine the amount of active molecule in the protein preparation. When the specificity test is based on interaction between molecules this measurement can be performed by surface plasmon resonance (SPR) using the "calibration-free concentration analysis" (CFCA) method, which has been implemented in different SPR instruments available commercially. More details can be found in:

- The importance of correct protein concentration for kinetics and affinity determination in structure-function analysis. *Pol E. J Vis Exp* ***2010; 37:2–8****.*

[15] **Endotoxin content**

Limulus Amebocyte Lysate (LAL) assay, utilizing blood from the Horseshoe crab (*Limulus polyphemus*), is the standard assay for endotoxin quantification and can be purchased by several commercial vendors.

- Biochemical principle of Limulus test for detecting bacterial endotoxins. Iwanaga S. *Proc Jpn Acad, Ser B, Phys Biol Sci* ***2007; 83:110–119****.*

[16] **Additives use to stabilize folding and prevent aggregation**

- Effect of Additives on Protein Aggregation Hamada, H. et al. *Current Pharmaceutical Biotechnology,* **2009, 10: 400-407**
- Molecular level insight into intra-solvent interaction effects on protein stability and aggregation. Shukla, D. et al. *Adv Drug Deliv Rev* ***2011;*** **doi:10.1016/j.addr.2011.06.014**
- Lebendiker M. http://wolfson.huji.ac.il/purification/Protocols/Additives_Folding.html

[17] **Example: optimization of buffer condition by automated DLS**

DLS set-up that allows processing a large number of samples in plate format, has simplified buffer condition screening. Buffer matrices for multi-parametric screening of pH, salinity, buffer nature, additives and co-factors can be generated by hand or using simple robotics. One approach is to dilute samples in the different buffer, classically ten times with a final concentration of 1 mg/ml for a 10 kDa protein or 0.1 mg/ml for a 100 kDa protein. The homogeneity of the sample and the presence of aggregates can be assessed for each condition, and the optimal buffer composition can be selected, according to solubility parameters and the downstream application.

More details about this approach can be found in:

- Solubility at the molecular level: development of a critical aggregation concentration (CAC) assay for estimating compound monomer solubility. Wang J, Matayoshi E. *Pharm Res* ***2012; 29:1745–1754.***

[18] **Example: optimization of buffer condition by thermal shift assay**

Modern thermofluor and differential scanning fluorimetry (DSF) assays that allow processing a large number of samples are suitable methods for screening buffer conditions. Similarly, to automated DLS, buffer matrices can be generated.

In a thermal shift assay, thermal denaturation, i.e. unfolding is monitored by following the fluorescence of an environmentally sensitive probe upon heating of the sample. This probe can be an external dye (e.g. SYPRO orange in the thermofluor assay) or the protein's own tryptophan residues in DSF (intrinsic tryptophan fluorescence). Fluorescence of the probe will change with temperature, since the hydrophobicity of the probe's local environment changes during unfolding. Usually, a sigmoidal curve is obtained, where the inflection point represents the temperature at which 50% of the protein is unfolded, often called the T_m. Buffer conditions that stabilize the protein will lower the T_m.

More details about this approach can be found in:

- Optimization of protein purification and characterization using Thermofluor screens. Boivin S, Kozak S, Meijers R. Protein Expr Purif **2013; 91:192–206.**
- A thermal stability assay can help to estimate the crystallization likelihood of biological samples. Dupeux F, Röwer M, Seroul G, Blot D, Márquez JA. *Acta Crystallogr D Biol Crystallogr* ***2011; 67:915-9.***
- Thermal activation of the bovine Hsc70 molecular chaperone at physiological temperatures: physical evidence of a molecular thermometer. Leung S-M, Senisterra G, Ritchie KP, Sadis SE, Lepock JR, Hightower LE. *Cell Stress Chaperones* ***1996; 1:78-89***.
- Theory and applications of differential scanning fluorimetry in early-stage drug discovery. Gao, K., Oerlemans, R. & Groves, M.R. *Biophys Rev* **2020; 12, 85–104**. https://doi.org/10.1007/s12551-020-00619-2

[19] **Batch-to-batch consistency**

Use some of the methods listed above, e.g. spectroscopic techniques such as UV spectroscopy and circular dichroism are rapid and effective methods of to assess and compare the quality of various batches of the same protein.

Supplementary Note 2. Examples of protein quality assessment

Example 1 (SEC).

Protein: Human ASPP and iASPP protein domains produced in *E. coli*

Aim of the project: studying protein-peptide interactions

Problem: insufficient reproducibility

Resolutive protein quality analysis: SEC detected aggregation

Action: buffer optimization for purification and storage

New outcome: stable proteins

Reference: Amir A., van Rosmalen M., Mayer G., et al. (2015) Highly homologous proteins exert opposite biological activities by using different interaction interfaces. Sci. Rep. 5:11629

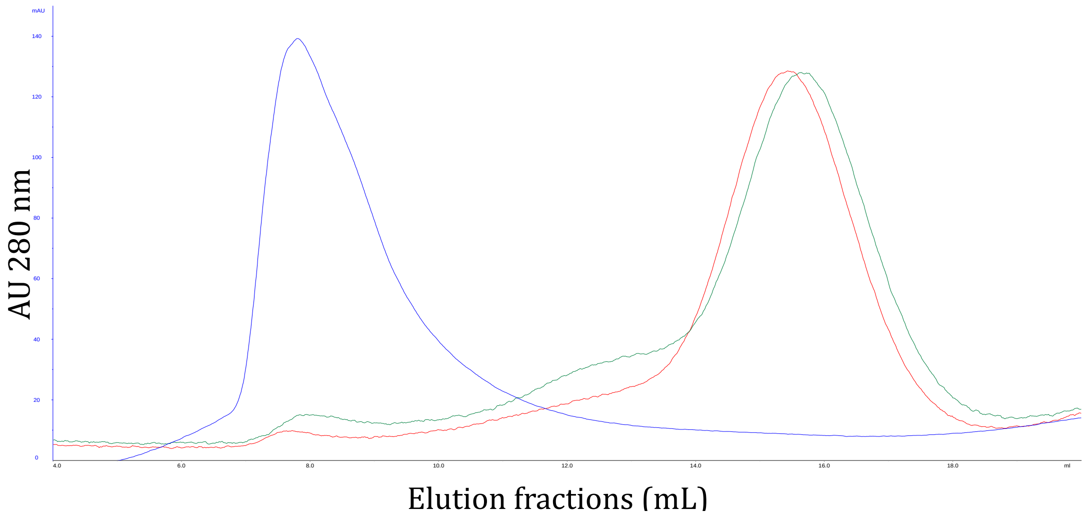

Supplementary Figure 8: IMAC-purified protein fractions analyzed by gel filtration.

Blue trace: sample in initial buffer.
Green trace: sample in buffer integrated with 0.5 M trehalose.
Red trace: sample processed with buffer integrated with 0.001% Tween 20

Example 2 (DLS).

Proteins: Human epsin and Sla2 N-term domains produced in *E. coli*

Aim of the project: structure determination of the endocytic adaptor protein complex ANTH and ENTH2 (AENTH)

Problem: protein aggregation which impaired crystallization and SAXS analysis

Resolutive protein quality analysis: DLS to identify the native 8:8 hetero dimers

Action: optimization of the PIP2 concentration

New outcome: stable oligomers suitable for crystallography

Reference: Garcia-Alai M.M., Heidemann J., Skruzny M., et al. (2018) Epsin and Sla2 form assemblies through phospholipid interfaces. Nature Commun. 9:328.

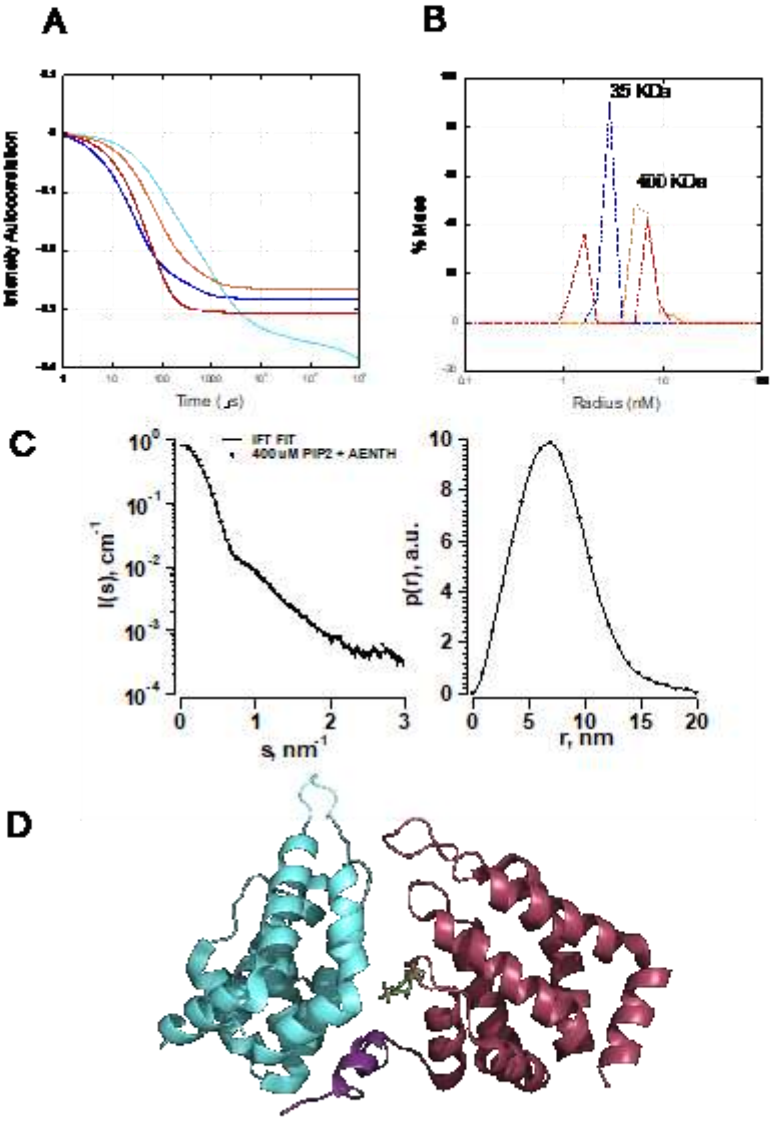

Supplementary Figure 9: A) DLS Autocorrelation functions of the endocytic adaptor protein complex ANTH and ENTH2 (AENTH) in the absence and presence of phosphatidylinositol 4,5-bisphosphate (PIP2); 0 uM (blue), 80 µM (cyan), 200 uM (orange) and 400 uM (red). B) DLS Percentage of mass as a function of the hydrodynamic radius (nm). C) SAXS analysis of the AENTH complex in the presence of 400 uM PIP2, experimental intensity (left panel). Distance distribution plot computed from the experimental data and normalized to the maximum value of unity (right panel). D) Crystal structure of the ENTH2 complex in the presence of 400 uM PIP2 (5ON7).

Example 3.

Proteins: Peptide from commercial sources

Aim of the project: Use of the peptide for interaction studies by ITC

Problem: The measured mass (7809 Da) of the synthetic peptide did not correspond to the expected mass (7550 Da)

Resolutive protein quality analysis: DLS to identify aggregates, MALDI mass spectrometry to confirm integrity and identity

Action: Re-synthesis of peptide

New outcome: Confirmation of the peptide identity

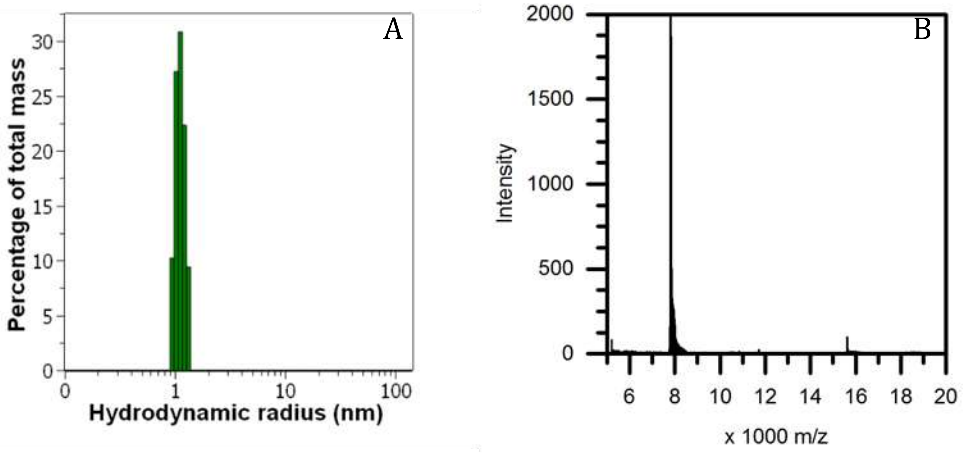

Supplementary Figure 10: Identification of wrong peptide sequence

A) DLS Percentage of mass as a function of the hydrodynamic radius (nm). B) Maldi-Tof intact mass analysis.

Unpublished data by courtesy of Patrick Walter, Institut Pasteur

Example 4.

Proteins: Proteins produced in and purified from *E. coli*.

Aim of the project: Proteins for structural determination using X-ray crystallography.

Problem: The measured mass (by top-down/intact MALDI-Tof MS) revealed a heterogeneous protein mass due to the mis-incorporation of lysine residues at the place of arginine (AGA) codons

Resolutive protein quality analysis: MALDI-Tof mass spectrometry to confirm integrity and identity

Action: Re-expression in and purification from *E. coli* (over-)expressing ArgU/dnaY tRNA (recognizing both AGG and AGA codons) from the pRARE plasmid

New outcome: Protein with correct/homogenous molecular mass

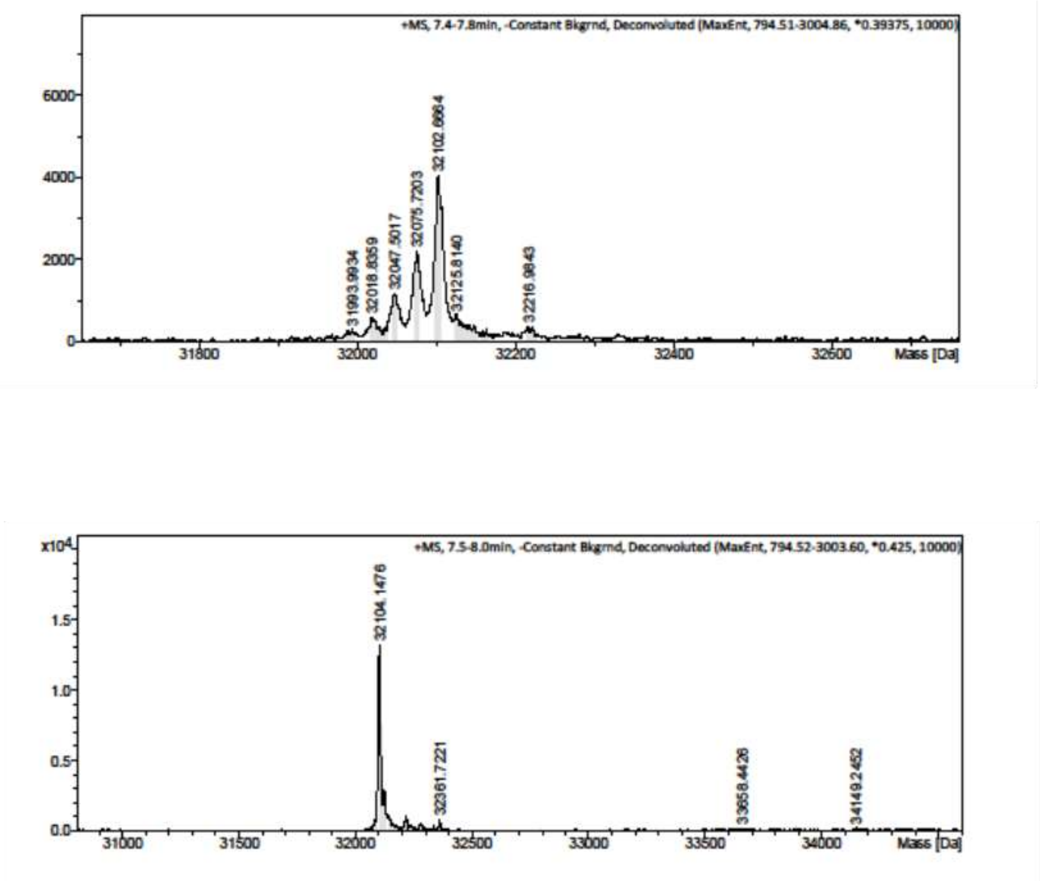

Supplementary Figure 11: Deconvoluted mass spectrum of protein grown in BL21-AI cells, note the multiple masses detected (Top) and deconvoluted mass spectrum of protein grown in BL21-AI cells supplemented with the pRARE plasmid encoding ArgU/dnaY tRNA (Bottom).

Unpublished data by courtesy of Sabine Suppmann and Nagarjuna Nagaraj, Biochemistry Core Facility at the Max-Planck Institute of Biochemistry, Munich.

Example 5.

Protein: produced in *E. coli*

Aim of the project: analysis of protein-DNA complex

Problem: In transmission electron microscopy (TEM) experiments in the presence of DNA, components failed to assemble into native large DNA-protein complexes but rather appeared as single particles (Figure C, left panel). The hetero-dimer showed no sign of impurities (as tested by SDS-PAGE, Fig A) but assessment of the sample by DLS indicated the presence of soluble aggregates (Fig. B left panel, note the broad main peak suggesting poly-dispersity and the aggregate peak indicated with an arrow)

Resolutive protein quality analysis: buffer optimization experiments were performed using DLS to assess sample dispersity

Action New outcome: the hetero-dimers remained mono-dispersed (Figure B, right panel, remark the narrower main peak with no aggregate peak visible)

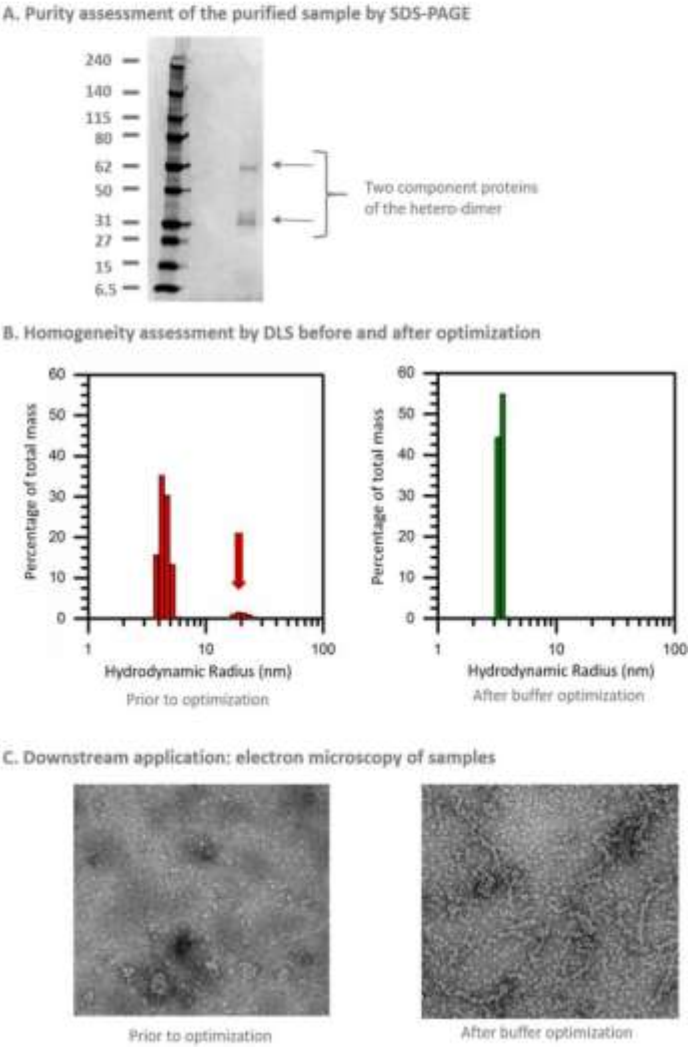

Supplementary Figure 12: Example of the use of DLS as a QC procedure for sample dispersity. A-SDS-PAGE of the hetero-dimeric complex; B-left panel, DLS data obtained with the sample in the original purification buffer (note the indicated aggregate peak); right panel, DLS data obtained with the sample in the optimized buffer; C-left panel, negatively–stained transmission TEM images obtained with the sample in the original purification buffer in the presence of DNA; right panel, negatively–stained TEM images obtained with the sample in the optimized buffer in the presence of DNA.

Unpublished data by courtesy of Chiara Rapisarda and Thomas Perry - Structure and function of bacterial nano-machines, Institut Européen de Chimie et Biologie, Bordeaux
